# Incidence and clinical relevance of cage subsidence in anterior cervical discectomy and fusion: a systematic review

**DOI:** 10.1007/s00701-018-3490-3

**Published:** 2018-02-21

**Authors:** Iris Noordhoek, Marvyn T. Koning, Wilco C. H. Jacobs, Carmen L. A. Vleggeert-Lankamp

**Affiliations:** 1 Department of Neurosurgery, Leiden University Medical Center, Albinusdreef 2, 2300 RC Leiden, the Netherlands; 2 Department of Hematology, Leiden University Medical Center, Leiden, the Netherlands

**Keywords:** Anterior discectomy, Fusion, Cage, Subsidence

## Abstract

**Background:**

The placement of intervertebral cages in anterior cervical discectomy (ACDF) supposedly maintains foraminal height. The most commonly reported cage-related complication is subsidence, although it is unknown whether a correlation between subsidence and clinical outcome exists.

**Aim:**

To assess the incidence and relevance of subsidence.

**Methods:**

Literature searches were performed in PubMed, MEDLINE, Embase, Web of Science, COCHRANE, and CENTRAL. The inclusion criteria were as follows: ≥ 20 patients, ADCF with cage, subsidence assessed, and primary data. Risk of bias was assessed using adjusted Cochrane checklists.

**Results:**

Seventy-one studies, comprising 4784 patients, were included. Subsidence was generally defined as ≥ 3-mm loss of height comparing postoperative intervertebral heights with heights at last follow-up. Mean incidence of subsidence was 21% (range 0–83%). Of all patients, 46% of patients received polyether-ether-ketone (PEEK) cages, 31% received titanium cages, 18% received cage-screw-combinations, and 5% received polymethyl-methacrylate (PMMA) cages. Patients treated with cage-screw-combinations had significantly less subsidence than patients treated with PEEK, titanium, or PMMA cages (15.1% vs. 23.5% vs. 24.9% vs. 30.2%; *p* < 0.001). Thirteen studies assessed clinical outcome in relation to subsidence; the majority did not find a significant correlation. Only four studies correlated subsidence to cage size and/or height; no correlation was established.

**Conclusions:**

Subsidence in ACDF with cages occurs in 21% of patients. The risk for subsidence seems lower using PEEK or titanium cages or adding screws. Whether subsidence affects clinical outcome is not satisfactorily evaluated in the available literature. Future studies on this correlation are warranted in order to establish the additional value of the interposition of a cage in ACDF.

## Introduction

Anterior cervical discectomy and fusion (ACDF) is a commonly used procedure to decompress cervical spinal nerves or the cervical medulla in case of predominant anterior compression. The discectomy provides decompression of the nervous tissue, while placing an intervertebral device, secured with or without anterior plating, maintains foraminal height, and ideally promotes eventual fusion. The intervention affords segmental stability and solid arthrodesis and carries minimal surgical risks [10, 14, 41].

When ACDF first became a well-accepted procedure in the 1950s, it was performed using autologous bone grafts obtained from the anterior iliac crest. However, this technique had several limitations, such as graft collapse or expulsion, pseudarthrosis, and significant donor-site morbidity. A remarkable number of cases showed subsidence, giving rise to loss of intervertebral height in the follow-up period [4, 7, 40]. Since then, cages have been introduced to replace the bone grafts, involving synthetic materials such as stainless steel, titanium, carbon fiber, polymethyl-methacrylate (PMMA), and polyether-ether-ketone (PEEK). In theory, the artificial cage types have the ability of restoration and preservation of disc height and lordosis, as well as the ability to prevent graft collapse or resorption [16, 27].

Although cages can differ in shape and material, they are all intended to maintain height and to add to immobilization of the degenerated motion segment. Ideally, cages serve as a scaffold to promote interbody fusion. However, side effects such as non-union, cage subsidence, and consequent kyphotic malalignment of the cervical spine are known to occur [38].

Since one of the goals of placing an intervertebral cage after anterior discectomy is to maintain foraminal height, it would be insinuated that cage subsidence is a particularly incapacitating complication, as this directly violates that goal. However, some surgeons perform this procedure without using an intervertebral device, and satisfactory results have been described as well [18, 30]. It is of interest to investigate whether the theoretical advantage of maintaining height is of clinical importance. Besides loss of height, cage subsidence can secondarily lead to pseudarthrosis, which causes (micro-)instability. Instability can lead to induction of bone formation by osteophytes, which can subsequently lead to recurring nerve root compression. Likewise, it is noteworthy to be informed on the correlation between subsidence and clinical result.

The primary objective of this systematic review is to determine the occurrence of cage subsidence after a cervical anterior discectomy with use of an intervertebral cage. Secondary objectives are to assess which dimensional aspects of the cage are related to subsidence, what qualifications the cages should meet in order to minimize the extent of this event, and whether there is a correlation between clinical outcome and cage subsidence.

## Materials and methods

### Data searches and study selection

**Fig. 1:**
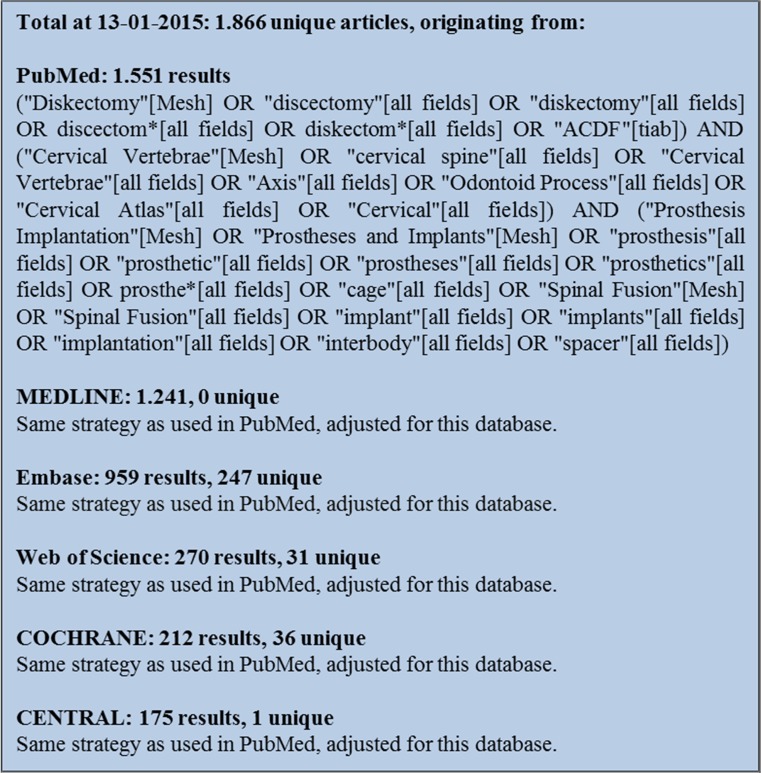
Search strings used for the data search in January 2015

In order to obtain all relevant literature, the electronic databases PubMed, MEDLINE, Embase, Web of Science, COCHRANE, and CENTRAL were searched in January 2015. The search strings presented in Fig. 1 were used. According to PRISMA guidelines, two of the authors (IN and MTK) individually and independently screened the articles for predefined inclusion criteria. These were stated as followed:

- The article was published in English or Dutch;
- The article was an original report presenting primary data;
- The article was published on or after 1 January 2000;
- The study had a minimum of 20 patients;
- The study reported a loss to follow-up of less than 20%;
- The study focused on the cervical spine (C2-Th1);
- The study presented patients undergoing anterior cervical discectomy and fusion with a cage;
- The study made an assessment of cage subsidence;
- The article was published in a peer-reviewed journal.

Only studies that the evaluators reached a consensus on were included. If needed, a third reviewer (CVL) was consulted.

### Quality assessment

To assess the quality of the selected studies, the studies were evaluated with the aid of an adjusted version of the checklist for cohort studies of the Dutch Cochrane Centre, presented in Table 1. The methodological requirements and objectives of these studies were closely evaluated. This was done individually and independently by two reviewers (IN and CVL). A third reviewer (WJ) was consulted in case of inconsistency. Studies were assessed on selection bias, for which a maximum of three points could be attributed; outcome bias, with a maximum of three points; follow-up bias, with a maximum of two points; and other bias, with a maximum of three points. In total, a study could be awarded a maximum of 11 points. Studies were then divided into a low (8–11 points), intermediate (6–7 points), or high (5 or less points) risk of bias group.

**Table 1:** Quality assessment checklist

Section	Award 1 point if
Selection bias (3 pts.)	
Goal and inclusion	Goal of the study is stated and study explicitly states the inclusion criteria
Selection of patients	Selective recruitment of patients can be ruled out
Patient characteristics	Study reports the age range and mean age and states the distribution of men and women
Outcome bias (3 pts.)	
Definition of subsidence	Definition of subsidence, classification, and radiological tools to measure subsidence were stated
Clinical outcome	Clinical outcome was systematically evaluated in correlation to subsidence
Preoperative status	Preoperative status was stated for comparison with postoperative status
Follow-up bias (2 pts.)	
Follow-up period	Follow-up range, period, and mean were given:
	• If yes and prospective study: 2 points
	• If yes and retrospective study: 1 point
	• If no, but loss to follow-up < 20%: 1 point
	• If too little information: 0 points
Other bias (3 pts.)	
Clinical evaluation	Evaluation was done independently from treating physician
Radiologic evaluation	Evaluation was done by an independent party, blinded to clinical results
Independence	Independence is explicitly stated, conflict of interest can be ruled out
Total (11 points)	

### Data extraction

All data from the included studies were analyzed and data regarding the following items were extracted:

- Number of participating patients;
- Mean incidence of subsidence;
- Distribution of patients over different cage types and correlation of cage type to subsidence;
- Clinical outcome and correlation to subsidence;
- Contact area and height of the cage and correlation to subsidence.

### Statistical analysis

Statistical analyses were done using Statistical Package for the Social Sciences (SPSS) version 22 (IBM, Armonk, NY, USA). Descriptive analysis was used to evaluate the data of this systematic literature review. To calculate the influence of the type of intervertebral cage on the incidence of subsidence, Fisher’s exact tests were used.

## Results

### Characteristics of included studies

Through our search, 1866 studies were identified. After matching these to our inclusion criteria, 71 studies were included. The most common grounds to exclude studies were as follows: studies appeared to be animal or cadaver studies, subsidence was not properly described, patient numbers were too small, and loss to follow-up was more than 20%. The 71 included studies stated a clear definition of subsidence and scored the incidence. Combining all these studies resulted in a cohort of 4784 patients, of whom 2216 received a PEEK cage, 1494 received a titanium cage, 833 received a combination of a cage and screws (cage-screw-combination, CSC), and 241 received a PMMA cage.

### Risk of bias

Twenty-eight studies were assessed to have a low risk of bias, 29 studies had an intermediate risk of bias, and 14 studies showed a high risk, mainly due to selection and follow-up bias (Table 2).

**Table 2:** Average amount of points in each risk of bias section when studies are assigned to a low, intermediate, or high risk of bias group

	Selection bias (3 pts.)	Outcome bias (3 pts.)	Follow-up bias (2 pts.)	Other bias (3 pts.)	Total (11 pts.)
Low (28)	2.0	2.9	1.6	2.1	8.7
Intermediate (29)	1.6	2.3	1.2	1.6	6.6
High (14)	1.1	1.9	0.8	0.8	4.6
Total (71)	1.7	2.4	1.3	1.6	7.0

### Incidence of cage subsidence

Subsidence was generally defined as ≥ 3 mm loss of height comparing the direct postoperative intervertebral height with the intervertebral height at the last follow-up moment. The mean incidence of subsidence was 21.1%, ranging from studies reporting 0% [1, 2, 12, 13, 15, 22, 26, 28, 29, 34, 37, 39] to 1 study reporting 83% [5]. When excluding the 14 studies with a high risk of bias from calculations, mean incidence of subsidence is 20.2% (range 0–83%).

### Correlation subsidence and clinical outcome

Clinical outcome was assessed in relation to subsidence in 13 studies [5, 8, 9, 11, 17, 20, 23–25, 33, 35, 45, 48]. Of these, 7 had a low risk of bias, 5 had an intermediate risk of bias, and 1 study had a high risk of bias. Of these 13 studies, 3 found a statistically significant correlation between a worse clinical outcome and the occurrence of subsidence [20, 23, 25]. The study by Kast et al. prospectively evaluated clinical outcome using the Odom scale [20]. The study by Kim et al. evaluated clinical outcome using the Odom scale as well, though in a retrospective manner from notes in the patients’ charts [23]. The study by Lee et al. evaluated clinical outcome through a visual analogue scale (VAS) for neck and arm pain. In the subsidence group, the VAS score for neck pain was 3.9 versus 2.5 in the non-subsidence group. The VAS score for arm pain was 3.7 in the subsidence group and 1.8 in the non-subsidence group [25]. The other 10 studies did not find a correlation between clinical outcome and the occurrence of subsidence. These studies generally used a larger variety of measures to evaluate clinical outcome and collected data prospectively in 4 studies [5, 9, 17, 33], and retrospectively in 6 studies [8, 11, 24, 35, 45, 48] (Table 3).

**Table 3:** Clinical outcome was assessed in correlation to subsidence in 13 studies

Reference	Bias score	*N* with subsidence/total (%)	Type of outcome measure	Study design	Correlation
Kast et al. [20]	10	15/52 (29)	Odom’s criteria	Prospective	Subsidence was correlated to worse outcome, *p* = 0.039
Kim et al. [23]	7	13/48 (27)	Odom’s criteria	Retrospective	Subsidence was correlated to worse outcome after 6 weeks (*p* = 0.021), 3 months (0.002), 6 months (*p* = 0.001), and 12 months (*p* = 0.05)
Lee et al. [25]	7	26/78 (33)	VAS for neck and arm pain	Retrospective	At last FU, the VAS score in the subsidence group was higher than in the non-subsidence group, *p* < 0.001
Chiang et al. [8]	6	8/56 (14)	Odom’s criteria	Retrospective	The satisfaction rate of subsidence cases seemed to be lower than that of the total population. However, this was not statistically significant
Brencke et al. [5]	8	66/80 (83)	VAS for neck pain, NDI, and PSI	Prospective	No correlation
Cho et al. [9]	8	1/60 (2)	Prolo scale for myelopathy and radiculopathy	Prospective	No correlation
Dogan et al. [11]	7	10/22 (47)	Nurick scale for myelopathy and Odom’s criteria	Retrospective	No correlation
Hwang et al. [17]	5	3/78 (4)	VAS for neck pain, Odom’s criteria	Prospective	No correlation
Klingler et al. [24]	7	39/107 (36)	VAS (not specified), NDI, SF-36, and PSI	Retrospective	No correlation
Orief et al. [33]	9	1/38 (3)	VAS for neck and arm pain and Odom’s criteria	Prospective	No correlation
Park et al. [35]	8	7/31 (23)	VAS (not specified), NDI, and Odom’s criteria	Retrospective	No correlation
Wu et al. [45]	10	10/57 (18)	JOA score for myelopathy, VAS for neck and arm pain	Retrospective	No correlation
Yoo et al. [48]	10	18/58 (31)	VAS for neck and arm pain, NDI, and Odom’s criteria	Retrospective	No correlation

*JOA* the Japanese Orthopedic Association, *VAS* visual analogue scale, *NDI* neck disability index, *PSI* patient satisfaction index, *SF-36* Short Form 36 Health Survey, *FU* follow-up

### Correlation subsidence and material of cage

The different types of cages that were used are PEEK, titanium, CSC, and PMMA. Of all patients, 46% were treated with PEEK cages, 31% with titanium cages, 18% with CSC, and 5% with PMMA cages. The mean incidence of subsidence in patients receiving a PEEK cage was 23.5%. Of all patients that received a titanium cage, 24.9% suffered from subsidence. In patients with a CSC, it was 15.1%; and in patients receiving PMMA cages, subsidence occurred in 30.2% (Table 4).

**Table 4:** Overview distribution of subsidence and patients over different cage types

	PEEK	Titanium	CSC	PMMA	Total
Studies (*n*)	44	27	16	6	71
Patients (*n*)	2216	1494	833	241	4784
Subsidence (%)	23.5	24.9	15.1	30.2	21.1
Average ROB	7.2	7.0	6.4	7.3	7.0

*ROB* risk of bias, *PEEK* polyether-ether-ketone, *CSC* cage-screw-combination, *PMMA* polymethyl-methacrylate

In patients treated with PMMA cages, there was a significantly higher incidence of subsidence than in patients treated with PEEK cages (*p* = 0.049) and CSC (*p* < 0.001). In patients treated with CSC, there was a significantly lower incidence of subsidence than in patients treated with PEEK, PMMA, or titanium cages (*p* < 0.001). There was no statistically significant difference between the incidence of subsidence between PEEK and titanium cages, nor between PMMA and titanium cages.

### Correlation subsidence and dimensional aspects of cage

Dimensional aspects of the cages were described in 16 studies [3, 6, 17, 19–21, 31, 32, 34, 36, 37, 42–44, 46, 47]. Only 4 of these assessed the dimensional aspects in relation to subsidence [6, 44, 46, 47], of which 1 study showed an increased risk of subsidence with a larger height of the cage (quote: “A cage size of 6.5 or 7.5 mm had a significantly higher risk of cage subsidence compared with 4.5 or 5.5 mm (p = 0.037).”) [46]. Another study showed a decreased risk of subsidence with a larger size of the contact surface of the cage (quote: “the use of 14 mm-diameter cages led to a significantly less risk for subsidence than using 12 mm-diameter cages (p = 0.034; odds ratio, 0.017)”) [47]. The other 2 studies did not find a correlation between the size or height of the cage and the occurrence of subsidence [6, 44]. These 4 studies all had a low risk of bias.

## Discussion

From the data presented in literature, it can be concluded that cage subsidence is a substantial side effect of intervertebral cage implantation in ACDF, with an overall incidence of 21%. However, the majority of studies correlating clinical outcome to the occurrence of subsidence did not find a negative clinical outcome with subsidence. The studies that did find a statistically significant correlation between worse clinical outcome and subsidence used the Odom scale for clinical outcome or a VAS score for pain. The Odom scale is a very rough scale (four options: very good, good, bad, and very bad) and in neither of the two studies was it indicated how the scale was dichotomized in order to draw conclusions. Furthermore, results were evaluated retrospectively in 2 of the 3 studies. Moreover, in all studies, the number of patients was limited (Table 3). In these three studies, on average, 50–70 patients were included, indicating that 10–14 patients per study suffered from subsidence (21%). Most studies that did not find a correlation between subsidence and clinical outcome collected their data retrospectively and used various types of outcome measures. The validity of the results in these studies is therefore debatable. In conclusion, it is neither possible to conclude that subsidence impacts the clinical outcome in a negative way, nor to conclude that subsidence does not impact clinical outcome at all.

Cages are introduced to maintain foraminal height to prevent the nerve root from being compressed in the neuroforamina after decompression. The foraminal height is frequently compromised after surgery, since the bulging disc is often accompanied by bony degeneration, leading to osteofytary rims with partial destruction of the uncinate process. Therefore, decompression of the cervical spinal nerve root usually comprises not only removal of disc tissue, but also removal of the bony rims, which damages the uncinate process. However, if the surgeon only removes the bulging disc tissue without damaging the uncinate process, the foraminal height can be maintained independently of the presence of an intervertebrate device.

Since none of the articles described the precise surgical procedure, no information on the integrity of the uncinate process is available. However, regardless of the integrity of the uncinate process, if loss of height of more than 3 mm (definition of subsidence) is not causing clinical symptoms, the urge to maintain foraminal height is debatable. Nevertheless, prevention of kyphosis and pseudarthrosis can still be regarded as beneficial effects of a cage, but these aspects have not been studied in the articles describing subsidence in ACDF.

A statistically significant correlation was found between the type of cage that was used and the occurrence of subsidence. CSCs seemed to have the least occurrence of subsidence, which only seems logical since the screws force the cage to be placed in the anterior cortical plane and additionally the screws themselves are placed cortically. The studies evaluating CSC generally had an intermediate risk of bias and were judged to be of adequate quality to base conclusions on. PMMA cages were found to have the highest occurrence of subsidence (30.2%), which is not only statistically higher than the general occurrence of subsidence (21.1%), but presumably also clinically relevant. The studies evaluating PMMA cages generally had a low risk of bias and are therefore valid studies to support the conclusion that PMMA cages should be avoided in ACDF when aiming to avoid subsidence. The differences between PEEK and titanium cages were not large enough to reach statistical significance, which leads to the conclusion that using either of those results in comparable outcome.

The prospective study by Yamagata et al. found that “a cage height of 6.5 or 7.5 mm had a significantly higher risk of cage subsidence compared with a height of 4.5 or 5.5 mm (p = 0.037)” [46]. This translates to larger heights of cages lead to more subsidence. This can be explained by the larger amount of stress on the vertebral endplates, which presumably results in subsidence. The retrospective study by Yang et al. found that “the use of 14 mm-diameter cages led to a significantly less risk for subsidence than using 12 mm-diameter cages (p = 0.034; odds ratio, 0.017)” [47]. This shows a decreased risk of subsidence with a larger size of the contact surface of the cage. However, it was not studied whether a diameter of for instance 18 or 22 mm leads to even fewer occurrences of subsidence, or whether perhaps there is an optimal diameter. The retrospective studies by van Jonbergen et al. [44] and Cabraja [6] only briefly mention that subsidence was not related to cage size. These studies did not discuss in detail what cage size or height they studied or how they performed their analyses. These 4 studies on cage size all had a low risk of bias. In summary, cage size and height cannot be excluded as risk factors for subsidence and these studies show interesting results; however, there is too little data to draw solid conclusions.

## Conclusion

Subsidence in ACDF using a cage occurs in 21% of patients. Based on the current review, the risk for subsidence seems to be lower when a PEEK or titanium cage is used, or when a cage with integrated screws is used. The correlation of subsidence with kyphosis or pseudarthrosis is not sufficiently addressed in the available literature. It is not satisfactorily evaluated whether subsidence affects clinical outcome. Future studies on these correlations are warranted in order to properly establish the value of the interposition of a cage in ACDF.
